# Dicoumarol: from chemistry to antitumor benefits

**DOI:** 10.1186/s13020-022-00699-0

**Published:** 2022-12-27

**Authors:** Vera L. M. Silva, Rita Silva-Reis, Alexandra Moreira-Pais, Tiago Ferreira, Paula A. Oliveira, Rita Ferreira, Susana M. Cardoso, Javad Sharifi-Rad, Monica Butnariu, Maria Alina Costea, Ioana Grozea

**Affiliations:** 1 LAQV-REQUIMTE, Department of Chemistry, University of Aveiro, 3810-193 Aveiro, Portugal; 2 Centre for the Research and Technology of Agro-Environmental and Biological Sciences (CITAB), Inov4Agro, University of Trás-os-Montes and Alto Douro, Vila Real, Portugal; 3 Inov4Agro—Institute for Innovation, Capacity Building and Sustainability of Agri-Food Production, University of Trás-os-Montes and Alto Douro (UTAD), 5000-801 Vila Real, Portugal; 4 Department of Veterinary Sciences, University of Trás-os-Montes and Alto Douro, Vila Real, Portugal; 5 Laboratory for Integrative and Translational Research in Population Health (ITR), Research Center in Physical Activity, Health and Leisure (CIAFEL), Faculty of Sports, University of Porto (FADEUP), 4200-450 Porto, Portugal; 6 Facultad de Medicina, Universidad del Azuay, Cuenca, Ecuador; 7 Life Sciences University “King Mihai I” from Timisoara, 300645 Calea Aradului 119, Timis, Romania

**Keywords:** Coumarins, Dicoumarol, Synthesis, Biological activity, Anticancer

## Abstract

Dicoumarol, a coumarin-like compound, is known for its anticoagulant properties associated with the ability to inhibit vitamin K, being prescribed as a drug for several decades. The pharmaceutical value of dicoumarol turned it into a focus of chemists’ attention, aiming its synthesis and of dicoumarol derivatives, bringing to light new methodologies. In recent years, several other bioactive effects have been claimed for dicoumarol and its derivatives, including anti-inflammatory, antimicrobial, antifungal, and anticancer, although the mechanisms of action underlying them are mostly not disclosed and additional research is needed to unravel them. This review presents a state of the art on the chemistry of dicoumarols, and their potential anticancer characteristics, highlighting the mechanisms of action elucidated so far. In parallel, we draw attention to the lack of in vivo studies and clinical trials to assess the safety and efficacy as drugs for later application.

## Introduction

Coumarins are a large family of compounds of natural or synthetic origin. In nature, coumarins are found in plants and microorganisms [1, 2]. Their structural variety and remarkable pharmacological activities, like antioxidant [3, 4], anti-inflammatory [5, 6], anticancer [7, 8], cardioprotective [9, 10], antimicrobial [11], antibacterial [12, 13], and enzymatic inhibition properties [14–18] are responsible for their top position in the field of natural products and medicinal chemistry [19, 20].

Dicoumarol, also known as bis-hydroxycoumarin, represents a good example of the benefits from the interaction of Western and traditional medicines. It was extracted and isolated for the first time in 1940 by Karl Link, from *Melilotus officinalis* (L.) Pall fungi [21]. This plant, commonly known as “yellow melilot” or “medicinal sweet clover,” is a species of the Fabaceae family and is widely distributed from Asia to Europe [22] and used as a medicine for centuries. Ancient Egyptians consumed tea made from this plant to treat earaches and intestinal worms. Also, this has been employed as a traditional Chinese herb with purifying and detoxifying properties [21]. In general, the beneficial blood-related qualities of *M. officinalis*, including lowering inflammation and increasing blood flow [23], are closely associated with coumarins, as they represent major compounds of this species. In this context, the isolation and recognition of the anticoagulant properties of dicoumarol led to its synthesis and of other derivatives having the same basic chemical structure.

Dicoumarol is well recognized for its anticoagulant and anti-inflammatory effects, and these properties have been reviewed by other authors [21, 24]. For the last years, this has also been highlighted to exert other important bioactive effects [21, 24]. To further understand these, it is crucial to discuss the chemistry of the compound in relation to its biological properties. Additionally, the compound’s anticancer effects are only briefly discussed, being important to distinguish the different mechanisms involved. Thus, this review summarizes the current level of knowledge about the chemistry of dicoumarols, their potential anticancer properties and the currently established mechanisms of action, for future medical applications.

## General characterization of dicoumarol

### Structure of dicoumarol

**Fig. 1:**
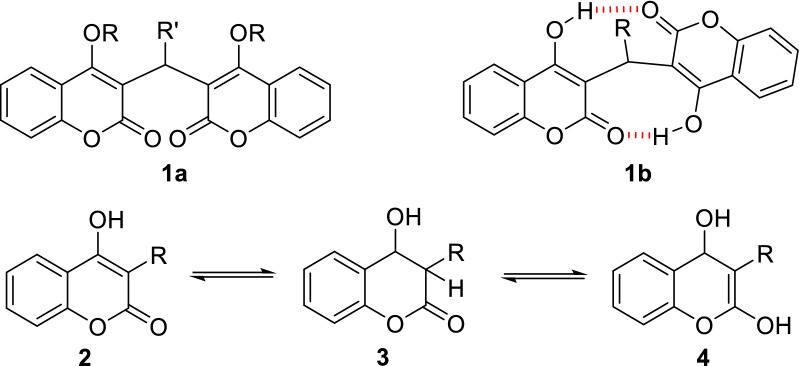
Structures of dicoumarol **1a**,**b** and tautomers of 4-hydroxycoumarin **2**–**4**

Chemically, dicoumarol **1**, a 3,3′-methylenebis-(4-hydroxycoumarin) (**1a**,**b**, R=R′=H), consists of two cyclic β-ketoesters linked by a methylenic bridge (Fig. 1). The parent compound is 4-hydroxycoumarin (**2**, R=H), which can be represented as one of three tautomeric structures **2**, **3** or **4** (Fig. 1). Several studies have demonstrated that coumarin form **2** is the main tautomer both in the solid state and in solution in polar solvents [25, 26]. Likewise, dicoumarols are often discussed because of their special molecular structures that may contain two intramolecular O–H$\cdots$O hydrogen bonds (**1b**) and distinct biological properties depending on the type of substituents on the central methylene linkage. A possible relationship between such hydrogen-bonded structure and the antimicrobial and antioxidant activities of dicoumarols is frequently suggested by different authors [27]. Still, for most publications cited in this review the structure shown for the synthesized dicoumarol derivatives is the one that corresponds to structure **1a**.

### Synthesis of dicoumarol

Due to the relevance of dicoumarol in the pharmaceutical field, the synthesis of this compound and its derivatives has been of major interest to chemists. Although there are some reports on the biosynthesis of dicoumarols by microorganisms that can use *o*-coumaric acid as the carbon source [28], and about their synthetic preparation starting from salicylic acid and formaldehyde [29], dicoumarols are generally synthesized by reaction of 4-hydroxycoumarin with different aldehydes in a 2:1 ratio. The position 3 in 4-hydroxycoumarin ring is highly activated, because of the influence of electron-donating hydroxy group and electron-withdrawing effects of carbonyl oxygen atom at the second position. There is a conjugation of π electrons from the double bond and the lone electron pairs in the oxygen atom. These two factors make the position 3 of coumarin ring very reactive for Michael addition reactions. In fact, the most common method for dicoumarols’ synthesis is the domino Knoevenagel–Michael reaction of 4-hydroxycoumarin with aldehyde derivatives and there have been developed several protocols for this reaction, that will be discussed in “Catalyst-free synthesis and synthesis using homogeneous catalysts” and “Synthesis using biocatalysts” sections.

#### Catalyst-free synthesis and synthesis using homogeneous catalysts

There are some publications on the synthesis of dicoumarols in the presence of 1,8-diazabicyclo[5.4.0]-undec-7-ene (DBU) as non-nucleophilic and strong tertiary amine base [30], in phosphoroxychloride in dry DMF [31], in refluxing ethanol and acetic acid [27, 32–35], under microwave irradiation in ethanol or solvent-free conditions [32], in the presence of diethylaluminum chloride (Et_2_AlCl) in dichloromethane [36], in the presence of catalytic amount of piperidine in ethanol [37] or other polar solvents like methanol, dimethylformamide or dimethyl sulfoxide at room temperature [38], and using triethylamine in methanol or sodium ethoxide in ethanol in the presence of cyanogen bromide [39]. It was also published some uncatalyzed one-pot synthesis of dicoumarols under conventional or microwave thermal solvent-free conditions, which is a simple, practical, and environmentally benign method to obtain dicoumarols in excellent yields [30, 40, 41] as well as the catalyst-free one-pot synthesis in water under ultrasounds irradiation at ambient temperature [42] or the “on solvent” reaction using minimal amount of boiling propanol without any catalyst [43]. In 2012, Mallik and coworkers developed an “on water” methodology for the synthesis of dicoumarols at 95 °C for 4–5 h. In their study, they observed a significant reduction of reaction time from 4–5 h to 0.5 h, if an electrolyte was added to the reaction. Therefore, several dicoumarol derivatives were synthesized in very good yields (83–96%) in aqueous NaCl (5 M) solution at 95 °C [44]. Aiming to overcome some drawbacks of previously cited methods such as the use of toxic reagents, solvents or catalysts, high temperature, long reaction time, and tedious workup procedures, other protocols have been developed for this reaction (Table 1). Among these protocols, several use Lewis acids, like molecular iodine [45], MnCl_2_ [46], Zn(Proline)_2_ [47] and InCl_3_ under microwave irradiation [48] or sulfated titania (TiO_2_/SO_4_^2−^) that behaves as Lewis and Bronsted acid [49] as catalysts. Inorganic acid salts like tris(hydrogensulfato)boron [B(HSO_4_)_3_] [50], and salts of transition metals such as ruthenium(III) chloride hydrate (RuCl_3_·*n*H_2_O) [51] have also been employed. With the increasing use of ionic liquids in organic synthesis, some methods have been developed using [bmim][BF_4_] [52], SO_3_H-functionalized ionic liquids [53], Brønsted acidic ionic liquids such as the 3-methyl-1-(4-sulfonic acid)butylimidazolium hydrogen sulfate [MIM(CH_2_)_4_SO_3_H][HSO_4_] under solvent-free conditions [54], tetramethylguanidium acetate ([TMG][Ac]) [55], choline hydroxide [56], poly(4-vinylpyridine)-supported ionic liquid ([P_4_VPy-BuSO_3_H]Cl-X(AlCl_3_)) [57, 58], 1,4-diazabicyclo[2.2.2]octane (Dabco)-based ionic liquid catalysts ([Dabco-H][AcO]) [58] or *N*-methylpyrrolidonium zinc chloride (Hnmp/ZnCl_3_)-based Brønsted–Lewis acidic ionic liquids [59]. Heteropoly acids, namely phosphotungstic acid [60], and phase transfer catalysts or surfactants like tetrabutylammonium bromide (TBAB) [61] and sodium dodecyl sulfate (SDS) [62] have also been used. Chemically modified glycerols, obtained by treatment of glycerol with agents that can react with hydroxy groups, possess physicochemical properties different from the parent glycerol and have been used as catalysts. For example, propane-1,2,3-triyl tris(hydrogen sulfate) (PTTH), prepared by addition of chlorosulfonic acid to glycerol at 0 °C, was employed in the synthesis of dicoumarols in water or solvent-free conditions at 80 °C with success [63]. The highly solving action of this catalyst on the reactants promotes their interaction and reactivity, and the carbonyl carbon of the aldehyde is activated by the intermolecular hydrogen bonding with the hydroxy groups of PTTH. In addition, the formed intermediates are stabilized by several types of complexations and hydrogen bonding with the hydroxy groups of PTTH. Most of the methods presented in Table 1 are of simple execution and are efficient since for most of them dicoumarol derivatives are obtained with very high yield and the reaction scope is broad because a wide range of aliphatic, and diversely substituted aromatic and heteroaromatic aldehydes, containing both electron-donating and electron-withdrawing substituents, are well-tolerated. Some of these methods are also considered environmentally friendly, especially those that use water as solvent or solvent-free neat conditions, thus avoiding the use of volatile and toxic organic solvents. In most of these methods [47, 49, 51, 52, 54–60, 62, 63] the catalyst can be recovered and reused, although with progressive loss of catalytic activity and consequent gradually decrease of reaction yields.

**Table 1:** Synthetic methods of dicoumarols using different types of catalysts
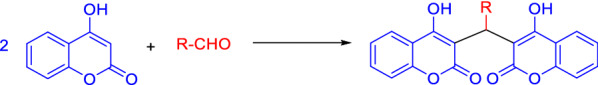

Type of catalyst	Catalyst	Conditions	Dicoumarol derivatives R (yields)	Refs.
Lewis acids	I_2_	I_2_ (10 mol%), H_2_O, 100 °C, 1 atm, 20–34 h	Ph (97%), 2-HOC_6_H_4_ (98%) 3-O_2_NC_6_H_4_ (94%), 4-ClC_6_H_4_ (93%) 4-O_2_NC_6_H_4_ (95%), 4-HOC_6_H_4_ (98%) 4-MeOC_6_H_4_ (99%), –CH–CH–C_6_H_4_ (92%) Furan-2-yl (93%), thiophen-2-yl (91%) Indol-3-yl (95%), 3,4-piperonyl (96%)	[45]
	MnCl_2_	MnCl_2_ (10 mol%), H_2_O, 100 °C, 20–40 min	MeCH=CH– (95%), Ph (99%) 2-HOC_6_H_4_ (93%), 4-ClC_6_H_4_ (99%) 4-HOC_6_H_4_ (95%), 4-MeOC_6_H_4_ (97%) 4-O_2_NC_6_H_4_ (99%), furan-2-yl (96%) thiophen-2-yl (95%), indol-2-yl (94%)	[46]
	Zn(Proline)_2_	Zn(Proline)_2_ (5 mol%), H_2_O, reflux, 5–9 min	Ph (92%), 2-ClC_6_H_4_ (96%) 2-HOC_6_H_4_ (92%), 3-O_2_NC_6_H_4_ (93%) 4-ClC_6_H_4_ (94%), 4-HOC_6_H_4_ (96%) 3-MeO-4-HOC_6_H_3_ (91%) thiophen-2-yl (93%) 5-Me-thiophen-2-yl (92%) pyridin-4-yl (91%)	[47]
	InCl_3_	InCl_3_ (10 mol%), H_2_O, MW, 110 °C, 15–20 min	Ph (92%), 4-ClC_6_H_4_ (91%), 4-FC_6_H_4_ (93%) 4-HOC_6_H_4_ (90%), 4-MeC_6_H_4_ (85%) 4-MeOC_6_H_4_ (87%), 4-O_2_NC_6_H_4_ (96%) 4-HO-3-MeOC_6_H_3_ (89%) 3,4-(HO)_2_C_6_H_3_ (85%) 4-HO-3,5-(MeO)_2_C_6_H_2_ (86%)	[48]
Lewis and Bronsted acids	Sulfated titania^a^ (TiO_2_/SO_4_^2−^)	(TiO_2_/SO_4_^2−^) (15%), H_2_O, 80 ºC, 12–30 min	H (95%), Et (88%), Ph (92%), Pr (90%) ^i^Pr (90%), Me-CH=CH– (92%) 2-HOC_6_H_4_ (95%), 2-MeOC_6_H_4_ (85%) 2-O_2_NC_6_H_4_ (90%), 3-BrC_6_H_4_ (91%) 3-ClC_6_H_4_ (92%), 3-HOC_6_H_4_ (84%) 3-MeOC_6_H_4_ (88%), 3-O_2_NC_6_H_4_ (90%) 4-BrC_6_H_4_ (94%), 4-ClC_6_H_4_ (96%) 4-HOC_6_H_4_ (96%), 4-MeC_6_H_4_ (89%) 4-MeOC_6_H_4_ (92%), 4-O_2_NC_6_H_4_ (88%) 4-OH-3-MeOC_6_H_3_ (89%) 3-OH-4-MeOC_6_H_3_ (85%) 3,4-(MeO)_2_C_6_H_3_ (90%) 3,4,5-(MeO)_3_C_6_H_2_ (94%) 4-BnO-3-MeOC_6_H_2_ (82%) 4-CNC_6_H_4_ (96%), 4-Me_2_NC_6_H_4_ (89%) Ph-CH=CH– (86%), naphthalen-1-yl (85%) Furan-2-yl (90%)	[49]
Inorganic acid salts	B(HSO_4_)_3_	B(HSO_4_)_3_ (0.3 equiv) (1:1, H_2_O–EtOH), 70 °C, 3–6 min	Ph (86%), 3-MeOC_6_H_4_ (81%) 3-O_2_NC_6_H_4_ (82%), 4-BrC_6_H_4_ (95%) 4-ClC_6_H_4_ (92%), 4-NCC_6_H_4_ (86%) 4-FC_6_H_4_ (88%), 4-MeC_6_H_4_ (87%) 4-MeOC_6_H_4_ (83%), 4-O_2_NC_6_H_4_ (98%) 4-Cl-3-O_2_NC_6_H_3_ (88%)	[50]
Transition metals salts	RuCl_3_·*n*H_2_O	RuCl_3_·*n*H_2_O (5 mol%), H_2_O, 80 °C, 25–35 min	Et (75%), Ph (84%), 4-ClC_6_H_4_ (85%) 4-NCC_6_H_4_ (95%), 4-MeOC_6_H_4_ (92%) 3-(PhO)-C_6_H_4_ (90%), 2-Cl-6-FC_6_H_3_ (92%) 3,4-(F_2_)C_6_H_3_ (90%) 2-HO-3-MeOC_6_H_3_ (84%) 3,4-(MeO)_2_C_6_H_3_ (84%), indol-3-yl (90%) 2-O_2_NC_6_H_4_CH=CH– (90%)	[51]
Ionic liquids	[bmim]BF_4_	[bmim]BF_4_ (4 equiv) 60–70 °C, 2–3 h	Ph (84%), 3-ClC_6_H_4_ (84%) 4-BrC_6_H_4_ (87%), 4-ClC_6_H_4_ (91%) 4-MeC_6_H_4_ (83%), 4-MeOC_6_H_4_ (87%) 4-O_2_NC_6_H_4_ (84%), Me_2_CH– (77%) Ph-CH=CH– (82%), furan-2-yl (83%) pyridin-2-yl (81%)	[52]
	SO_3_H-functionalized ILs based on benzimidazolium cation	[PSebim][OTf]^b^ (10 mol%), 70 °C, 2–3 h	Ph (95%), 3-BrC_6_H_4_ (94%) 3-ClC_6_H_4_ (94%), 4-BrC_6_H_4_ (95%) 4-ClC_6_H_4_ (96%), 4-MeC_6_H_4_ (93%) 4-MeOC_6_H_4_ (93%), 4-O_2_NC_6_H_4_ (96%) 4-(H_2_C=CH_2_)C_6_H_4_ (92%)	[53]
	[MIM(CH_2_)_4_SO_3_H][HSO_4_]	[MIM(CH_2_)_4_SO_3_H] [HSO_4_] (15 mol%), 80 °C, 18–30 min	Ph (92%), 2-ClC_6_H_4_ (88%) 2-O_2_NC_6_H_4_ (86%), 3-ClC_6_H_4_ (89%) 3-O_2_NC_6_H_4_ (89%), 4-ClC_6_H_4_ (93%) 4-MeC_6_H_4_ (90%), 4-MeOC_6_H_4_ (89%) 4-O_2_NC_6_H_4_ (96%)	[54]
	Tetramethyl guanidium acetate ([TMG][Ac])	[TMG][Ac] (0.75 mmol), rt, 0.5–4.5 h	Ph (96%), 2-HOC_6_H_4_ (90%) 2-O_2_NC_6_H_4_ (92%), 3-O_2_NC_6_H_4_ (87%) 4-BrC_6_H_4_ (96%), 4-ClC_6_H_4_ (88%) 4-FC_6_H_4_ (93%), 4-F_3_CC_6_H_4_ (91%) 4-MeC_6_H_4_ (90%), 4-MeOC_6_H_4_ (86%) 4-O_2_NC_6_H_4_ (99%), pyridin-4-yl (87%) 3-MeO-4-HOC_6_H_3_ (84%), furan-2-yl (95%) (98%)	[55]
	Choline hydroxide	ChOH (40%)^c^, 50 °C, 1–3 h	H (quantitative), Ph (99%) 2-HOC_6_H_4_ (99%), 2-O_2_NC_6_H_4_ (75%) 3-O_2_NC_6_H_4_ (93%), 4-BrC_6_H_4_ (94%) 4-ClC_6_H_4_ (99%), 4-FC_6_H_4_ (99%) 4-F_3_CC_6_H_4_ (96%), 4-HOC_6_H_4_ (94%) 4-MeC_6_H_4_ (94%), 4-MeOC_6_H_4_ (95%) 4-HO-3-MeOC_6_H_3_ (93%), furan-2-yl (98%) 4-O_2_NC_6_H_4_ (89%), pyridin-4-yl (81%) (95%)	[56]
	[P_4_VPy-BuSO_3_H]Cl-X(AlCl_3_)^d^	IL (0.07 mmol), toluene, 90 °C, 0.5–0.9 h	Ph (95%), 2-HOC_6_H_4_ (90%) 3-ClC_6_H_4_ (96%), 3-O_2_NC_6_H_4_ (96%) 4-ClC_6_H_4_ (93%), 4-MeC_6_H_4_ (94%) 4-MeOC_6_H_4_ (92%), 4-HOC_6_H_4_ (90%) 4-O_2_NC_6_H_4_ (96%), furan-2-yl (91%) Thiophen-2-yl (91%), pyridin-2-yl (91%) Ph–CH_2_–CH_2_ (92%), CH_3_CH_2_CH_2_– (92%) Ph-CH=CH– (93%)	[57]
	[Dabco-H][AcO]	[Dabco-H][AcO] (10 mol%), H_2_O, 80 °C, 2–15 min	*n*-Pr (99%), Ph (98%), 2-BrC_6_H_4_ (98%) 3-BrC_6_H_4_ (98%), 4-BrC_6_H_4_ (99%) 4-ClC_6_H_4_ (99%), 4-MeC_6_H_4_ (96%) 4-MeOC_6_H_4_ (98%), 4-O_2_NC_6_H_4_ (99%) 2,4-Cl_2_C_6_H_3_ (96%), naphthalen-2-yl (99%) thiophen-2-yl (98%), furan-2-yl (98%)	[58]
	Hnmp/ZnCl_3_	(Hnmp/ZnCl_3_) (20 mg), 100 °C, 30–50 min	Ph (97%), 3-ClC_6_H_4_ (86%) 3-MeOC_6_H_4_ (90%), 3-O_2_NC_6_H_4_ (81%) 4-ClC_6_H_4_ (90%), 4-HOC_6_H_4_ (81%) 4-MeC_6_H_4_ (88%), 4-MeOC_6_H_4_ (93%) 4-O_2_NC_6_H_4_ (86%), 2,4-(MeO)_2_C_6_H_3_ (78%) pyridin-4-yl (90%)	[59]
Heteropoly acids (HPAs)	Phosphotungstic acid	HPA (15 mmol%), H_2_O, 80 °C, 14–25 min	Ph (93%), 2-ClC_6_H_4_ (95%) 2-HOC_6_H_4_ (98%), 2-O_2_NC_6_H_4_ (96%) 3-O_2_NC_6_H_4_ (94%), 4-ClC_6_H_4_ (93%) 4-FC_6_H_5_ (98%), 4-HOC_6_H_4_ (98%) 4-MeOC_6_H_4_ (99%), 4-O_2_NC_6_H_4_ (95%) 2,4-Cl_2_C_6_H_3_ (92%), 2,6-Cl_2_C_6_H_3_ (98%) –CH=CH–C_6_H_4_ (98%), 3,4-piperonyl (96%) Indol-3-yl (95%), thiophen-2-yl (91%) Furan-2-yl (93%), 4-F_3_CC_6_H_4_ (98%) 4-Me_2_C_6_H_3_ (90%), 3,4-(MeO)_2_C_6_H_3_ (90%)	[60]
Phase transfer catalysts and surfactants	Tetrabutyl ammonium bromide (TBAB)^e,f^	TBAB (10 mol%), H_2_O, 100 °C	Ph (92%), 3-ClC_6_H_4_ (87%) 4-BrC_6_H_4_ (88%), 4-ClC_6_H_4_ (95%) 4-MeC_6_H_4_ (92%), 4-MeOC_6_H_4_ (84%) 4-O_2_NC_6_H_4_ (91%), Ph–CH=CH– (82%) 3,4-(MeO)_2_C_6_H_3_ (87%), Me_2_CH– (82%) 3,4,5-(MeO)_3_C_6_H_2_ (84%), piperonyl (88%) furan-2-yl (88%), pyridin-2-yl (90%) 4-(Me)_2_CHC_6_H_4_ (91%)	[61]
	Sodium dodecyl sulfate (SDS)	SDS (20 mol%), H_2_O, 60 °C, 2.30–3.0 h	Ph (90%), 2-MeC_6_H_4_ (84%) 3-ClC_6_H_4_ (92%), 3-O_2_NC_6_H_4_ (95%) 4-BrC_6_H_4_ (91%), 4-ClC_6_H_4_ (93%) 4-FC_6_H_4_ (94%), 4-MeC_6_H_4_ (97%) 4-(Me)_2_NC_6_H_4_ (94%), 4-MeOC_6_H_4_ (97%) 4-O_2_NC_6_H_4_ (98%), 3,4-(MeO)_2_C_6_H_3_ (98%)	[62]
Modified glycerols	Propane-1,2,3-triyl tris(hydrogen sulfate) (PTTH)	PTTH (0.03 mol%), 80 °C, H_2_O, 7–10 min^g^ or solvent-free, 5–8 min	H (80%), Ph (90%), 2-ClC_6_H_4_ (85%) 3-O_2_NC_6_H_4_ (95%), 4-ClC_6_H_4_ (90%) 4-HOC_6_H_4_ (85%), 4-MeC_6_H_4_ (90%) 4-MeOC_6_H_4_ (85%), 4-O_2_NC_6_H_4_ (95%) 3,4-(MeO)_2_C_6_H_3_ (85%), Ph-CH=CH– (85%) furan-2-yl (80%)	[63]

^a^The catalyst possesses as many Lewis acid sites as Bronsted acid sites. The superacidity is due to the Bronsted acidic hydroxy groups attached to Ti atom, being generated by interaction with water. The acidity is enhanced by the generation of Lewis acid sites due to—I effect exerted by attached SO_4_^2−^ ions to Ti atoms. These factors promote the overall reaction by activating the aldehydes and the Michael acceptor

^b^[PSebim][OTf]=1-ethyl-3-(3-sulfopropyl)-benzimidazolium trifluoromethanesulfonate

^c^Amount of catalyst is 1.5 equiv. relative to the aldehyde

^d^[P_4_VPy-BuSO_3_H]Cl-X(AlCl_3_) is poly(4-vinylpyridine-co-1-sulfonic acid butyl-4-vinylpyridinium)chloroaluminate, a supported ionic liquid with both Lewis and Brønsted acid sites

^e^This catalyst was also used in the synthesis of dicoumarol derivatives under solvent-free conditions. The reaction times were slightly shortened in neat conditions, and the yields were also slightly lower

^f^TBAF was found to be equally effective as catalyst

^g^Yields for the reaction in water indicated in the table

**Fig. 2:**
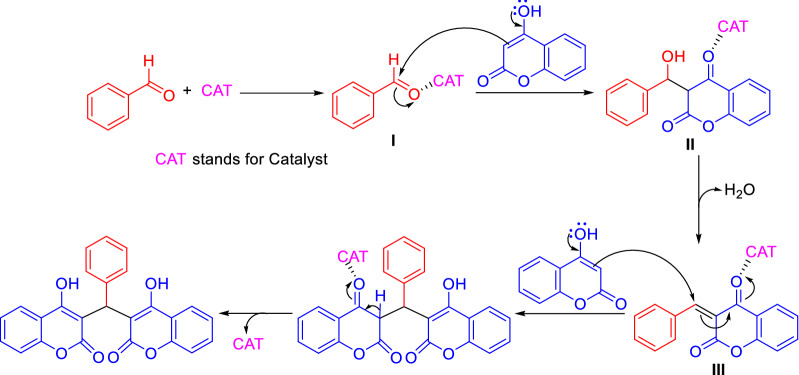
Generic mechanism for the formation of dicoumarol by domino Knoevenagel–Michael reaction of 4-hydroxycoumarin with aldehyde derivatives

Regarding the reaction mechanism, this is similar for most of the methods reported in Table 1. The first step consists in the activation of the aldehyde through the binding of the catalyst (CAT) with the carbonyl oxygen to increase the electrophilicity of the carbonyl carbon of the aldehyde. Then a nucleophilic addition of 4-hydroxycoumarin to the catalyst–aldehyde complex (**I**) followed by elimination of water gives intermediate (**II**) which is further activated by the binding of the catalyst to the carbonyl oxygen (**III**) thus facilitating the reaction with another molecule of 4-hydroxycoumarin. Finally, an enolization occurs with concomitant formation of the dicoumarol (Fig. 2).

#### Synthesis using heterogeneous catalysts

As a result of the ongoing research for simpler, greener, and more efficient methods for the synthesis of dicoumarols, novel methodologies have been developed taking advantage of the introduction of more advanced tools in organic synthesis. In this sense, heterogeneous catalysts, including nanoparticles, and magnetic nanoparticles have gained much importance in recent years due to economic and environmental considerations. These catalysts are generally less expensive, highly reactive, eco-friendly, easy to handle, recover and reuse.

**Scheme 1:**
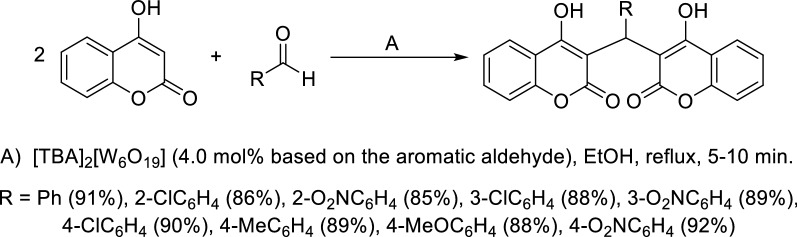
Synthesis of dicoumarols catalyzed by [TBA]_2_[W_6_O_19_]

Davoodnia reported a highly efficient and fast method for the synthesis of dicoumarols, in ethanol, using tetrabutylammonium hexatungstate [TBA]_2_[W_6_O_19_] as a green and reusable heterogeneous catalyst (Scheme 1) [64]. For other solvents like methanol, chloroform, or acetonitrile, and in solvent-free conditions only moderate yields were achieved. Since the catalyst is not soluble in hot ethanol it can be easily recovered.

**Scheme 2:**
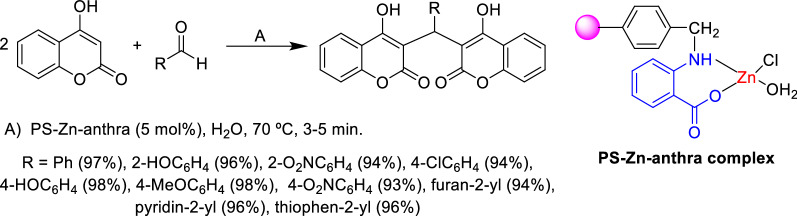
Synthesis of dicoumarol derivatives in water catalyzed by PS-Zn-anthra complex

Another methodology was described for the synthesis of dicoumarol derivatives in water utilizing a polystyrene functionalized zinc anthranilic acid complex (PS-Zn-anthra complex) as a non-toxic and reusable Lewis acid catalyst (Scheme 2) [65]. The main advantages of this methodology are operational simplicity, mild reaction conditions, almost quantitative yields, short reaction time, low loading of catalyst, and the possibility to recycle the catalyst for five times without appreciable loss of its activity.

**Scheme 3:**
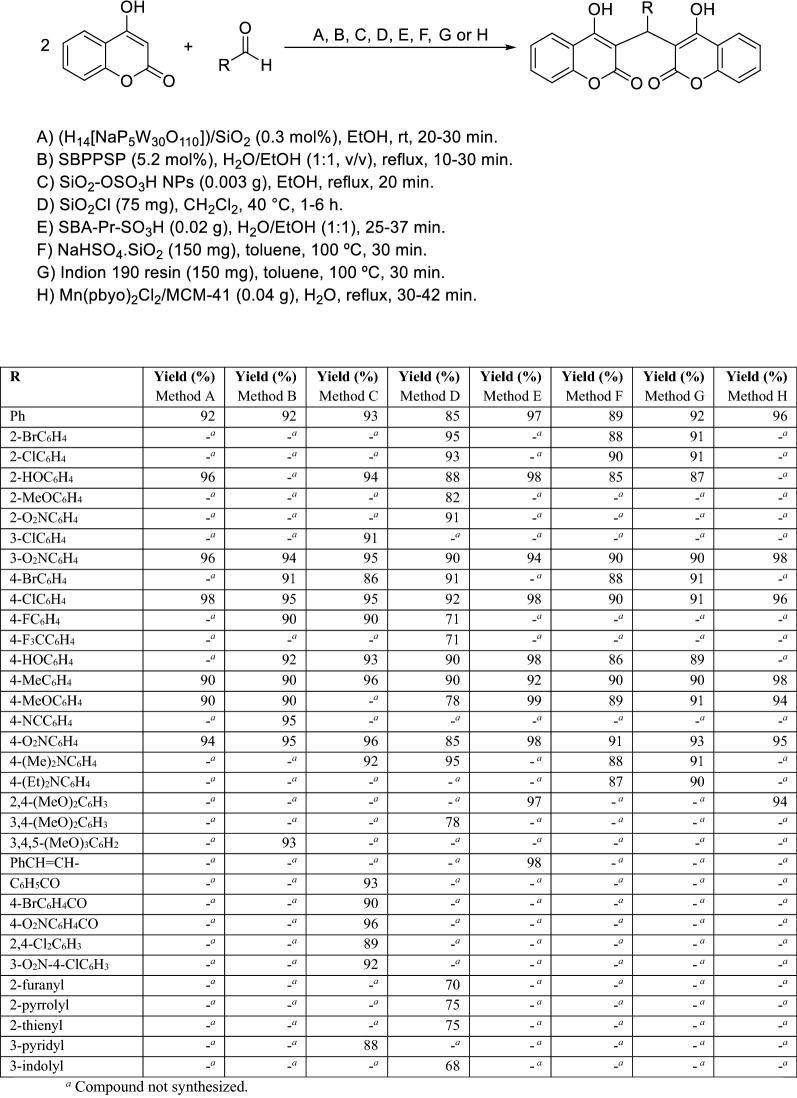
Synthesis of dicoumarols using silica-based heterogeneous catalysts

In 2010, Heravi et al. reported the synthesis of dicoumarols by one-pot domino Knoevenagel-type condensation/Michael reaction between 4-hydroxycoumarin and aromatic aldehydes in the presence of Preyssler type H_14_[NaP_5_W_30_O_110_] immobilized into SiO_2_ nanoparticles. By comparison with other catalysts and with the non-supported Preyssler type catalyst, the authors have shown that only 0.3 mol% of (H_14_[NaP_5_W_30_O_110_])/SiO_2_ can efficiently catalyze the reaction in ethanol and its reusability is another advantage (Scheme 3) [66]. Two years later, Niknam and coworkers published the use of silica-bonded *N*-propylpiperazine sodium *n*-propionate (SBPPSP) as a recyclable basic catalyst for the synthesis of dicoumarols in aqueous ethanol (1:1 v/v) under reflux conditions (Scheme 3) [67]. In 2013, another method was reported using silica sulfuric acid nanoparticles (SiO_2_–OSO_3_H NPs) as a solid acid catalyst under reflux in ethanol (Scheme 3) [68]. In the same year, Karimian et al. described the use of nano silica chloride (nano SiO_2_Cl) as an efficient, chemoselective and recyclable catalyst for facile and simple condensation of 4-hydroxycoumarin with aromatic and heteroaromatic aldehydes into dicoumarols in dry dichloromethane (Scheme 3) [69]. In turn, Ziarini et al. reported the synthesis of dicoumarols in the presence of sulfonic acid functionalized mesoporous silica SBA-15 as an efficient nanoporous heterogeneous solid acid catalyst using a mixture of H_2_O/EtOH (1:1) as solvent (Scheme 3) [70]. Later, other protocols for dicoumarols’ synthesis were developed using the silica-supported sodium hydrogen sulfate (NaHSO_4_·SiO_2_) and Indion 190 resin (Scheme 3) [71]. First, a methylidene-2*H*-chromene-2,4(3*H*)-dione intermediate is formed by the nucleophilic addition of 4-hydroxycoumarin to the activated aldehydes in the presence of NaHSO_4_·SiO_2_ or Indion 190 resin. Then, Michael addition of methylidene-2*H*-chromene-2,4(3*H*)-dione with a second unit of 4-hydroxycoumarin followed by dehydration afforded the expected dicoumarol [71]. The authors investigated the reusability of the catalyst. After completion of the reaction the catalyst was separated by filtration, washed with hexane, and dried. The activated catalyst was used for two more subsequent cycles with no loss of activity [71]. Since the discovery of MCM-41 (Mobil Composition of Matter No. 41) by Mobil Corporation scientists [72], these silica-based materials have been employed as efficient catalysts and catalyst supports. MCM-41 possesses several unique properties, including high surface area, uniform and tunable pore sizes, excellent physicochemical stability, and modifiable surfaces. However, its direct use as a catalyst is difficult due to a lack of sufficient acidity [73]. However, the functionalization of MCM-41 with transition metals, especially by manganese (Mn), leads to effective catalysts. One example, that has been used in the synthesis of dicoumarols is the solid acid catalyst Mn(pbyo)_2_Cl_2_/MCM-41 containing the 2,2′-bipyridine-6,6′-dionate (Scheme 3) [73].

**Scheme 4:**
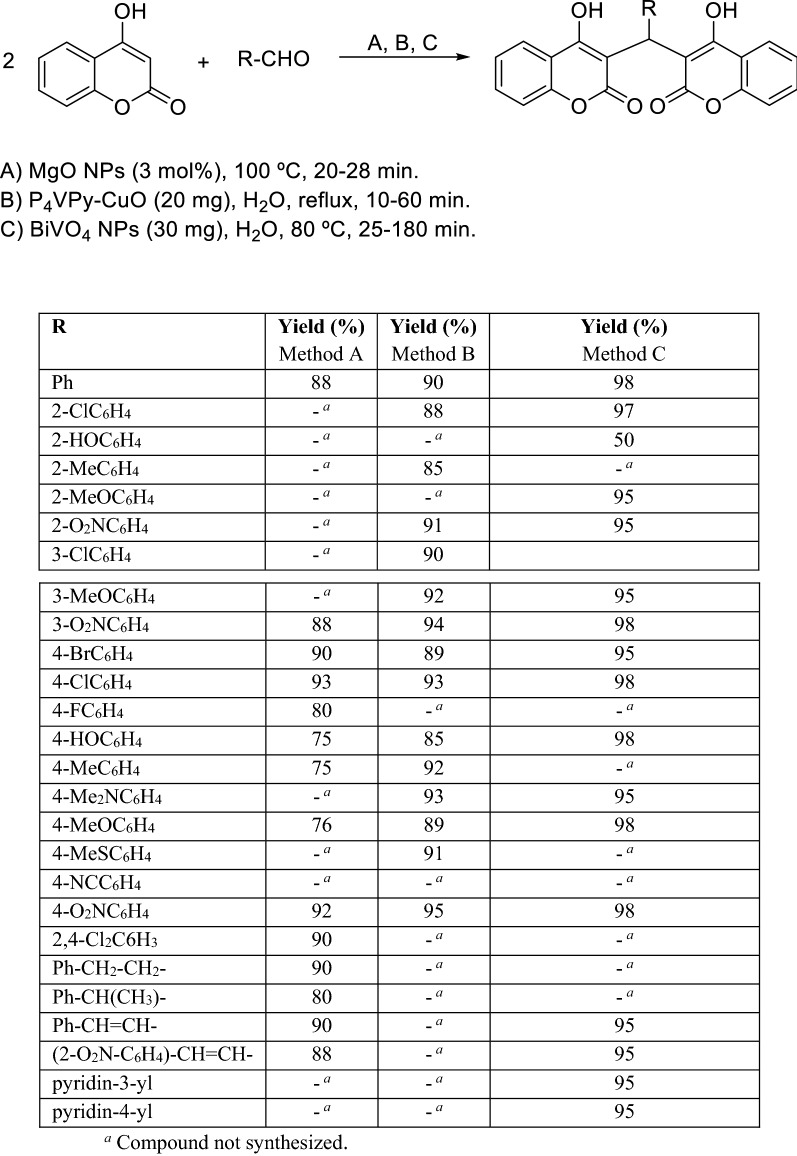
Synthesis of dicoumarols using MgO, PVP-CuO and BiVO_4_ nanocatalysts

Among the various nano catalysts magnesium oxide (MgO) finds out extensive application as heterogeneous catalyst. In 2014, Safaei-Ghomi and coworkers described magnesium oxide nanoparticles as an efficient, available, and cheap heterogeneous catalyst for the synthesis of dicoumarols in solvent free conditions (Scheme 4) [74]. Poly(vinylpyridine) (PVP) is an appropriate polymer for immobilization of nanoparticles because of the strong affinity of the pyridyl group to metals and its ability to make hydrogen bonds with polar species. In addition, PVP can interact electrostatically in quaternized or protonated forms with charged surfaces and therefore a variety of different PVP-supported reagents have been designed. In 2016, Shirini and coworkers reported the use of P_4_VPy–CuO-NPs as an effective catalyst for the synthesis of dicoumarols (Scheme 4) [75]. For the same purpose, 1 year later, bismuth vanadate nanoparticles (BiVO_4_-NPs) have also been used as an efficient and reusable nano-catalyst (Scheme 4). In the presence of this catalyst, yields of dicoumarols (95–98%) were superior compared to BiVO_4_ alone (75–95%) for a shorter reaction time [76].

**Scheme 5:**
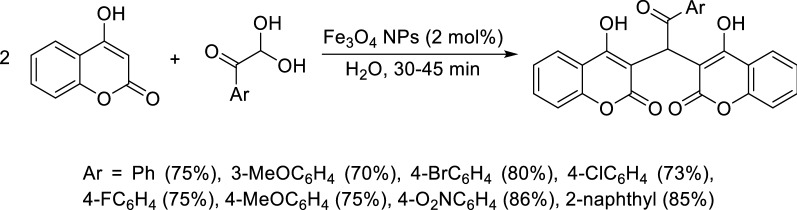
Synthesis of dicoumarol via the one-pot condensation of 4-hydroxycoumarin with aryl glyoxals

Another example is the use of iron oxide nanoparticles (Fe_3_O_4_ NPs) as magnetically recyclable and safe catalyst for the green synthesis of dicoumarols via the one-pot condensation of 4-hydroxycoumarin with aryl glyoxals on water (Scheme 5) [77]. Catalyst loadings can be as low as 2 mol% to give high product yield. Other advantages of this method are the avoidance of toxic organic solvents, and simple work-up procedure.

**Fig. 3:**
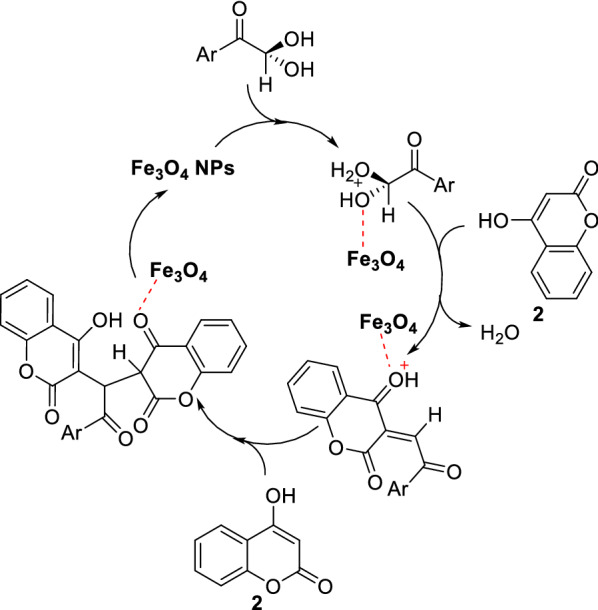
Plausible mechanism for the synthesis of dicoumarols from 4-hydroxycoumarin and aryl glyoxals in the presence of iron oxide nanoparticles

Mechanistically, in the presence of Fe_3_O_4_ NPs an active leaving group produced on aryl glyoxal by a Lewis acid such as iron(II, III) may be a reasonable start for the Knoevenagel condensation with 4-hydroxycoumarin (**2**). Subsequently, 1,4-addition of next 4-hydroxycoumarin (**2**) to the formed α,β-unsaturated ketone gave the target molecule. This mechanism also shows the recyclability of Fe_3_O_4_ NPs (Fig. 3) [77].

**Scheme 6:**
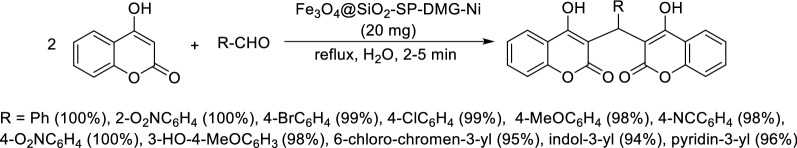
Synthesis of dicoumarols from 4-hydroxycoumarin in presence of Fe_3_O_4_@SiO_2_-SP-DMG-Ni(II) nanoparticles in water at reflux

The combination of water as a solvent with the use of immobilized catalysts that can be recovered and reused is even more desirable. A green synthesis of dicoumarol through reaction of 4-hydroxycoumarin with various aldehydes was achieved, in excellent yields and high rates, by using Ni-dimethylglyoxime complex immobilized on functionalized Fe_3_O_4_ by a post-grafting process (Fe_3_O_4_@SiO_2_-SP-DMG-Ni(II) NMPs) as a superparamagnetic catalyst (Scheme 6) [78]. This catalyst proved to be thermally stable, and efficient for this synthesis and was conveniently recovered using an external magnetic field and reused for subsequent reactions at least seven times without a significant decrease in catalytic activity.

Regarding the reaction mechanism, the Fe_3_O_4_@SiO_2_-SP-DMG-Ni(II) NMPs behave as a Lewis acid catalyst and activate the carbonyl group of the aldehyde, allowing immediate Knoevenagel condensation between 4-hydroxycoumarin and benzaldehyde. Then, water elimination gives Michael acceptor intermediate that undergoes a Michael addition with another 4-hydroxycoumarin and after enolization, the dicoumarol is obtained [78].

**Scheme 7:**
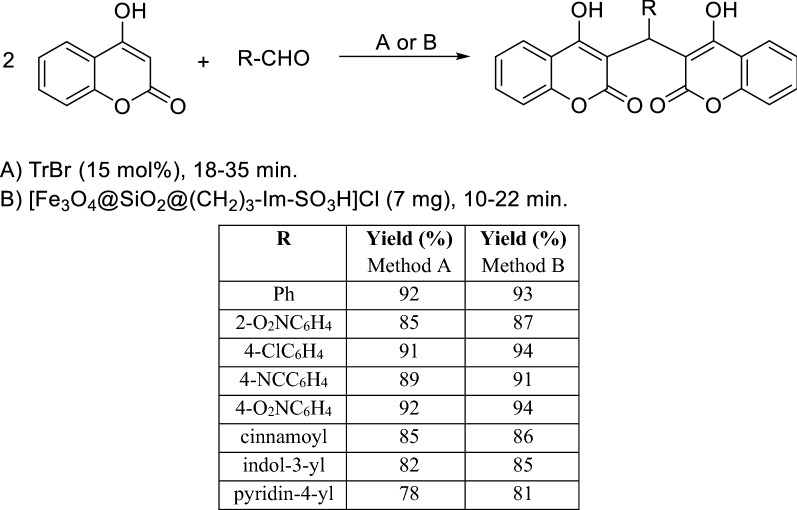
Synthesis of dicoumarol catalyzed by TrBr or [Fe_3_O_4_@SiO_2_@(CH_2_)_3_-Im-SO_3_H]Cl

Some recent works have described the synthesis of dicoumarols catalyzed by organocatalysts. For example, the use of trityl bromide (TrBr or Ph_3_CBr) as a neutral organocatalyst for the synthesis of dicoumarols in mild and solvent-free conditions was reported and the performance of this catalyst was found to be comparable to that of the nano-magnetic catalyst [Fe_3_O_4_@SiO_2_@(CH_2_)_3_-Im-SO_3_H]Cl (Scheme 7) [78].

**Scheme 8:**
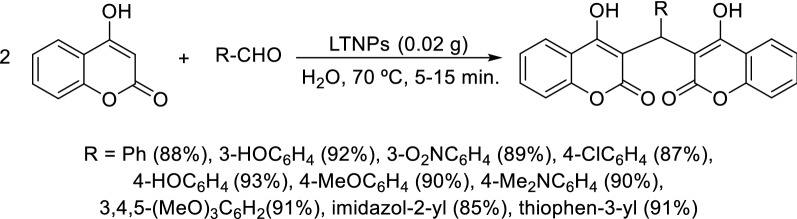
Synthesis of dicoumarol derivatives catalyzed by l-Tyrosine loaded nanoparticles

A work published in 2015, has demonstrated that the encapsulation of l-tyrosine organocatalyst into nanoparticles increases its ability to catalyze organic reactions [79]. In fact, l-tyrosine loaded nanoparticles (LTNPs) were used as catalyst in the synthesis of dicoumarol derivatives which were obtained in good yields (85–93%) in a short period of time (5–15 min) (Scheme 8). It was observed that with increment in the dielectric constant of protic solvents the catalyst gives higher yield in lesser time, which culminated in the choice of water as solvent. Increment in the catalyst surface area, very small catalyst loading, and easy separation of the catalyst are key advantages of this method.

#### Synthesis using biocatalysts

**Scheme 9:**
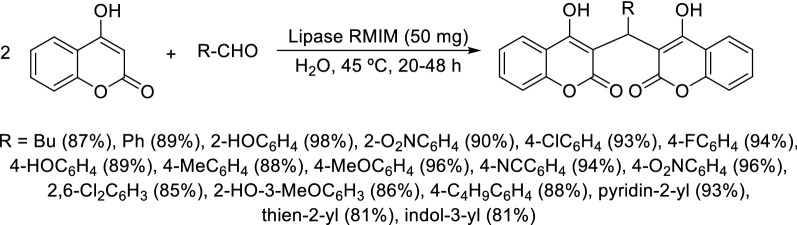
Synthesis of dicoumarols in water catalyzed by lipase RMIM

The use of biocatalysts in the Knoevenagel–Michael reaction for dicoumarol synthesis has been much less explored. Yet, a recent work was published using this type of catalysts. Hu and coworkers reported the use of the enzyme lipase from fungus *Rhizomucor miehei* (lipase RMIM) as a biocatalyst in the Knoevenagel–Michael cascade reaction of 4-hydroxycoumarin with aromatic, heterocyclic or aliphatic aldehydes to synthesize dicoumarol derivatives in water with excellent yields (81–98%) (Scheme 9) [80]. The mild reaction conditions, pure aqueous reaction system, broad substrate scope, recyclability of the catalyst, and operational simplicity make this an environmentally friendly process for dicoumarols’ synthesis. The first step of the reaction mechanism involves the interaction of the His-Asp residue of lipase with the hydroxyl group of 4-hydroxycoumarin to activate the compound for the Knoevenagel condensation with the aldehyde moiety, and the following steps involve a Mickael addition and enolization step as already described for the dicoumarols synthesis in other reaction conditions [80].

## Current and future potential medical applications

Dicoumarol is well-known for its pharmacological properties, mainly anticoagulant and anti-inflammatory activities. In fact, this was the first of the oral anticoagulants to be isolated and employed clinically. A vitamin K-dependent step in the hepatic synthesis of clotting factors II (prothrombin), VII, IX, and X is inhibited, leading to its anticoagulant activity. In particular, dicoumarol suppresses the carboxylation of glutamate residues in the N-terminal sections of vitamin K-dependent proteins, by impeding vitamin K reductase. This event limits the activation of vitamin K-dependent clotting factors, resulting in a decrease in prothrombin levels and consequently a decrease in clotting [81]. In this way, it acts as a vitamin K antagonist by inhibiting its bioavailability. It should be noted that dicoumarol acts to prevent the development of new thrombi but not to eliminate existing thrombi. This substance’s slow and irregular absorption can lead to gastrointestinal issues, main reasons why it has largely been replaced by warfarin [82].

Dicoumarol and associated anticoagulants are hydroxylated at the 4 position, which is necessary for potent anticoagulant action among other factors. Although several of the common plant coumarins do have very modest action when fed to animals in high doses, none of them have considerable clinical anticoagulant activity because they are not all replaced at this location [83]. Bleeding is the predominant adverse reaction of dicoumarol therapy. Hepatitis, hypersensitivity, and cholesterol embolization are a few more side effects that might manifest. Dicoumarol may be contraindicated alone or in combination with other thrombolytic drugs and clotting inhibitors [82]. Additionally, the European Medicined Agency recommended using dicoumarol for earaches and eye ulcers [22]. Currently, in the Netherlands, Poland, Austria, and Germany it is used as a medicine with antioxidant and anticoagulant activities, in the United Kingdom it is used to treat edema and for the circulation of the renal vein [84].

In addition to the well-recognized pharmacological properties of dicoumarol, it is known to have other activities of interest for future applications, including as anticancer (detailed in “Antitumor action of dicoumarol” section) and as antimicrobial agents. Dicoumarol was initially discovered as a rodenticide [85] and that said, several in vitro studies have explored its potential as an antimicrobial agent, namely antibacterial, antiviral, and anti-fungal. Dholariya et al. [86] conducted a study where they analyzed the antibacterial activity of dicoumarol and a series of its derivatives containing Cu^2+^ complexes. The antibacterial activity was exerted for the gram-negative bacterium (*Pseudomonas aeruginosa* ATCC25619 and *Escherichia coli* ATCC25922) and gram-positive bacterium (*Bacillus subtilis* ATCC11774 and *Streptococcus pyogenes* ATCC12384). Moreover, it also exerted antifungal activity against *Aspergillus niger* ATCC64958 and *Candida albicans* ATCC6602. Dicoumarol also exerted anti-tubercular activity for *Mycobacterium tuberculosis* (H37Rv and H37Ra strains) [86, 87], further demonstrating that this compound increased the sensitivity of anti-tuberculosis drugs (such as isoniazid and rifampicin) when it was present at a low concentration [87]. Dicoumarol inhibits human immunodeficiency virus-1 (*HIV-1*) gene replication by degrading Tat (a protein encoded by *HIV-1*) through inhibition of NAD(P)H:quinone oxidoreductase, allowing steady-state levels to maintain capacity in viral transcripts [88]. In the study performed by Kammari et al*.* [89], the authors validated its anti-HIV activity. They observed anti-HIV-1 activity using dicoumarol pyridine derivatives with potential activity against a topoisomerase IIβ kinase, which is present in HIV-1 viral lysate. In another study, four synthetics derivatives dicoumarols (DC, 2-PyDC, 3-PyDC and 4-PyDC) were synthesized and characterized. All compounds showed inhibition in four *Staphylococcus aureus* bacterial strains such as *S. aureus* ATCC 29213, methicillin-resistant *S. aureus* (MRSA XJ 75302), vancomycin-intermediate *S. aureus* (Mu50 ATCC 700699), and USA 300 (Los Angeles County clone, LAC). Antibacterial activity was demonstrated for a minimum inhibitory concentration between 16 and 64 μg/mL, with the dicoumarol derivative, 2-PyDC (2-Pyridinodicoumarol), showing the most potent antibacterial activity [90].

## Antitumor action of dicoumarol

Dicoumarol has been gaining interest in the cancer setting due to its action as an inhibitor of nicotinamide adenine dinucleotide phosphate (NAD(P)):(quinone acceptor) oxidoreductase 1 (NQO1), by competing with NAD(P)H at the pyridine nucleotide binding site [91]. NQO1 is a cytosolic flavoenzyme that catalyzes the two-electron reduction of several quinone substrates to give their corresponding hydroquinones using both NADH and NADPH as electron donors [92], therefore avoiding the generation of reactive oxygen species (ROS) by redox cycling and functioning as an effective superoxide scavenger [92]. The toxicity of dicoumarol against cancer cells seems to be mainly mediated by the mitochondrial production of ROS due to NQO1 inhibition (Table 2) [93]. Nonetheless, other mechanisms independent of NQO1 inhibition have also been uncovered.

**Table 2:** Anticancer effects of dicoumarol assessed in vitro and in vivo

In vitro studies				
	Cancer type	Cell line	Dicoumarol effects	Refs.
NQO1 inhibition-dependent actions	Pancreatic	MIA PaCa-2	↑ **Apoptosis and oxidative stress via NQO1 inhibition** ↑ Cytosolic cytochrome *c* and cleaved PARP ↑ Total glutathione and GSSG	[97]
	Colon	HCT116	↑ **Miltirone-induced apoptosis and mitochondrial damage via ↓ p53 stability due to NQO1 deficiency** ↑ Loss of mitochondrial mass ↓ Mitochondrial ROS	[98]
	Urogenital	RT112 253J LNCap	↑ **Cisplatin cytotoxicity** ↓ p53-p21 pathway ↑ JNK	[99]
		KK47	↑ **Doxorubicin cytotoxicity** ↓ p53-p21 pathway ↑ p38-MAPK activation ↑ Cleaved caspase-3 ↓ Mcl-1	[100]
NQO1 inhibition-independent actions	Breast	MCF-7	↓ **Chemoresistance via PSG1-TGFβ1-EMT pathway** ↓ PSG1 expression ↓ TGFβ1 activation ↓ N-cadherin, vimentin and fibronectin expression	[101]
	Renal	Caki	↑ **TRAIL-mediated apoptosis** ↑ Cleaved PARP ↓ Bcl-2, Mcl-1, and c-FLIP expression ↓ NF-κB and CRE transcriptional activity	[102]
	Myeloid leukemia	HL-60	↑ **Superoxide release** Inhibition of OXPHOS complexes II, III and IV Inhibition pyrimidines biosynthesis	[91]
	Ovarian	SKOV3	↓ **PDK1 kinase activity, aerobic glycolysis → OXPHOS, ↑ apoptosis, and ↓ cell viability** ↓ p-PDHA1 ↑ Glucose uptake ↓ Lactate production ↑ Cleaved caspase-3, cleaved PARP ↑ ROS ↓ Mitochondrial membrane potential	[103]

In vivo studies				
	Cancer type	Animal model	Dicoumarol effects	Refs.
NQO1 inhibition-dependent actions	Pancreatic	MIA PaCa-2 xenografts (athymic nude mice)	↓ Tumor volume growth ↑ Animals’ survival	[97]
NQO1 inhibition-independent actions	Ovarian	SKOV3 xenografts (BALB/c-nu mice)	**Inhibition of PDK1 activity and ↑ apoptosis** ↓ p-PDHA1 ↑ Cleaved caspase-3 and cleaved PARP ↑ TUNEL^+^ cells ↓ Tumor volume and weight	[103]

*5-FU* 5-fluorouracil, *c-FLIP* cellular FLICE-like inhibitory protein, *CRE* cyclic adenose monophosphate response elements, *EMT* epithelial–mesenchymal transition, *GSSG* glutathione disulfide, *JNK* c-Jun N-terminal kinase, *MAPK* mitogen-activated protein kinase, *NF-κB* nuclear factor kappa B, *NQO1* NAD(P):(quinone acceptor) oxidoreductase 1, *OXPHOS* oxidative phosphorylation, *PARP* poly (adenosine diphosphate-ribose) polymerase, *PDHA1* pyruvate dehydrogenase E1 component subunit alpha, *PDK1* phosphoinositide-dependent kinase-1, *PSG1* pregnancy specific beta-1-glycoprotein 1, *ROS* reactive oxygen species, *TRAIL* tumor necrosis factor-related apoptosis-inducing ligand, *TGFβ1* transforming growth factor beta 1, *TUNEL* terminal deoxynucleotidyl transferase dUTP nick end labeling

Since the pharmacological and biological effects of dicoumarol have been explored in several contexts, its derivatives have also been gaining interest by the scientific community; however, the information available about the antitumor action of such derivatives is scarce [94, 95]. For instance, a study showed that the dicoumarol derivative spindlactone A (SPL-A) sensitized endometrial cancer Ishikawa cells to tumor necrosis factor-related apoptosis-inducing ligand (TRAIL)-induced apoptosis through the downregulation of the expression of cellular FLICE-like inhibitory protein (c-FLIP), Bcl-2, Bcl-xl and Mcl-1 and the upregulation of p53 expression, cleaved poly-(adenosine diphosphate-ribose) polymerase (PARP) levels, and caspase activity [96]. The apoptotic process in these cells was probably triggered by an SPL-A-induced increase in intracellular ROS levels via the inhibition of NQO1 [96]. It would be important to unravel derivatives with clinical potential that do not exhibit the off-target effects of dicoumarol (e.g., increasing oxygen radical formation and inhibition of oxygen respiration in normal cells) [94].

### NQO1 inhibition-dependent actions

By inhibiting NQO1, dicoumarol triggered oxidative stress (given by the increased intracellular production of superoxide anion and glutathione disulfide (GSSG)) and apoptosis and also inhibited cell growth of human MIA PaCa-2 pancreatic cancer cells [97, 104]. Apoptosis of this cell line seemed to be mediated by a dicoumarol-induced increase in cytosolic cytochrome *c* and cleaved PARP levels in a dose- and time-dependent manner [97]. Mechanistically, when the mitochondrial membrane permeability is disrupted, cytochrome *c* is released from mitochondria to the cytosol where it complexes with apoptotic protease-activating factor (Apaf) to activate caspase-9, which in turn can activate downstream caspases, like caspase-3. Caspase-3 is cleaved into its active form, which cleaves its substrate PARP, which is a marker of apoptosis [105]. These effects were confirmed *in vivo *with dicoumarol decreasing tumor growth in nude mice inoculated with MIA PaCa-2 cells and increasing the overall survival of the animals [97].

Data suggest that the inhibitory action of dicoumarol in NQO1 may also have a synergistic effect with other compounds via a mechanism based on p53 stabilization through NQO1. Dicoumarol, by inhibiting NQO1, induces p53 degradation, inhibiting p53-dependent p21 induction, and consequently, enhancing the apoptotic response [100]. This mechanism has been observed in several studies. Pretreatment of human colon cancer HCT116 cells with dicoumarol promoted miltirone-induced mitochondrial damage and apoptosis, effects that may be possibly the result of the dicoumarol-induced decrease of p53 stability due to NQO1 inhibition [98]. Dicoumarol also sensitized urothelial cancer cells with wild-type p53 (functional p53 seems to be necessary for the synergistic effect) to doxorubicin through p38 activation, which was induced by the suppression of the p53–p21 pathway [100]. Similarly, it enhanced the cytotoxicity of cisplatin in urogenital cancer cells with wild-type p53 through the suppression of p53–p21 pathway, which led possibly to JNK activation and apoptosis [99]. Moreover, this has been shown to enhance gemcitabine cytotoxicity in high NQO1 activity cholangiocarcinoma cells [106]. The authors suggested that the mechanism of dicoumarol-induced cell killing was not mediated by mitochondrial impairment and formation of ROS but related to the suppression of the pro-survival response to chemotherapy [106]. The combined therapy of dicoumarol with anticancer drugs may provide a novel therapeutic option to overcome chemoresistance in cancer treatment.

### NQO1 inhibition-independent actions

Dicoumarol effects on cancer cells may also be independent of its inhibitory action on NQO1 and several mechanisms have been proposed: (i) impairment of mitochondrial functionality; (ii) decrease of pregnancy-specific beta-1-glycoprotein 1 (PSG1) levels; (iii) inhibition of heat shock protein 90 (Hsp90); (iv) downregulation of anti-apoptotic proteins; (v) inhibition of heat shock protein 90 (Hsp90); and (vi) inhibition of phosphoinositide-dependent kinase-1 (PDK1).

Dicoumarol was reported to inhibit mitochondrial oxidative phosphorylation (OXPHOS) complexes II, III and IV, to stimulate superoxide anion release by reversed electron flow at OXPHOS complex II, and to inhibit the biosynthesis of pyrimidine at the dihydroorotate dehydrogenase step, leading to the accumulation of S phase cells in human myeloid leukemia HL-60 cell line under conditions of complete depletion of NQO1 [91].

In estrogen receptor-negative breast cancer (*in vitro* and *in vivo*), dicoumarol counteracted the chemoresistance to taxane-anthracycline-based chemotherapy by targeting the PSG1 [101]. Specifically, it decreased PSG1 levels and extracellular secretion, which led to the decrease in the activation of the PSG1 downstream mediator transforming growth factor β (TGFβ)1 [101]. TGFβ1 is an important contributor to cancer progression, including chemoresistance, since it induces pro-proliferation signals, such as epithelial-mesenchymal transition (EMT) signal [101]. The expression of EMT hallmarks, namely N-cadherin, vimentin and fibronectin, were inhibited by dicoumarol [101].

It was also demonstrated that dicoumarol treatment sensitized TRAIL-mediated apoptosis since it downregulated Bcl-2 expression by inhibiting nuclear factor kappa B (NF-κB) and CRE transcriptional activities, and Mcl-1 and cellular FLICE-like inhibitory protein (c-FLIP) expressions at the post-translational level (possibly by affecting protein stability). TRAIL is a known inducer of apoptosis, an effect that seems to be specific for cancer cells; however, TRAIL resistance is observed in several cancers [102]. In this way, dicoumarol may be used as a potential sensitizer for the treatment of TRAIL-resistant renal cancer [102].

Dicoumarol also downregulated PTTG1/Securin expression (a cell cycle protein that is elevated in many tumor types) by inhibiting Hsp90 (whose activity is increased in all cancers) [107]. Hsp90 inhibition leads to the downregulation of signaling pathways involved in proliferation, such as phosphoinositide 3-kinase (PI3K)/AKT and Raf-1, and to the induction of cell death by activating intrinsic and extrinsic pathways of apoptosis [107].

**Fig. 4:**
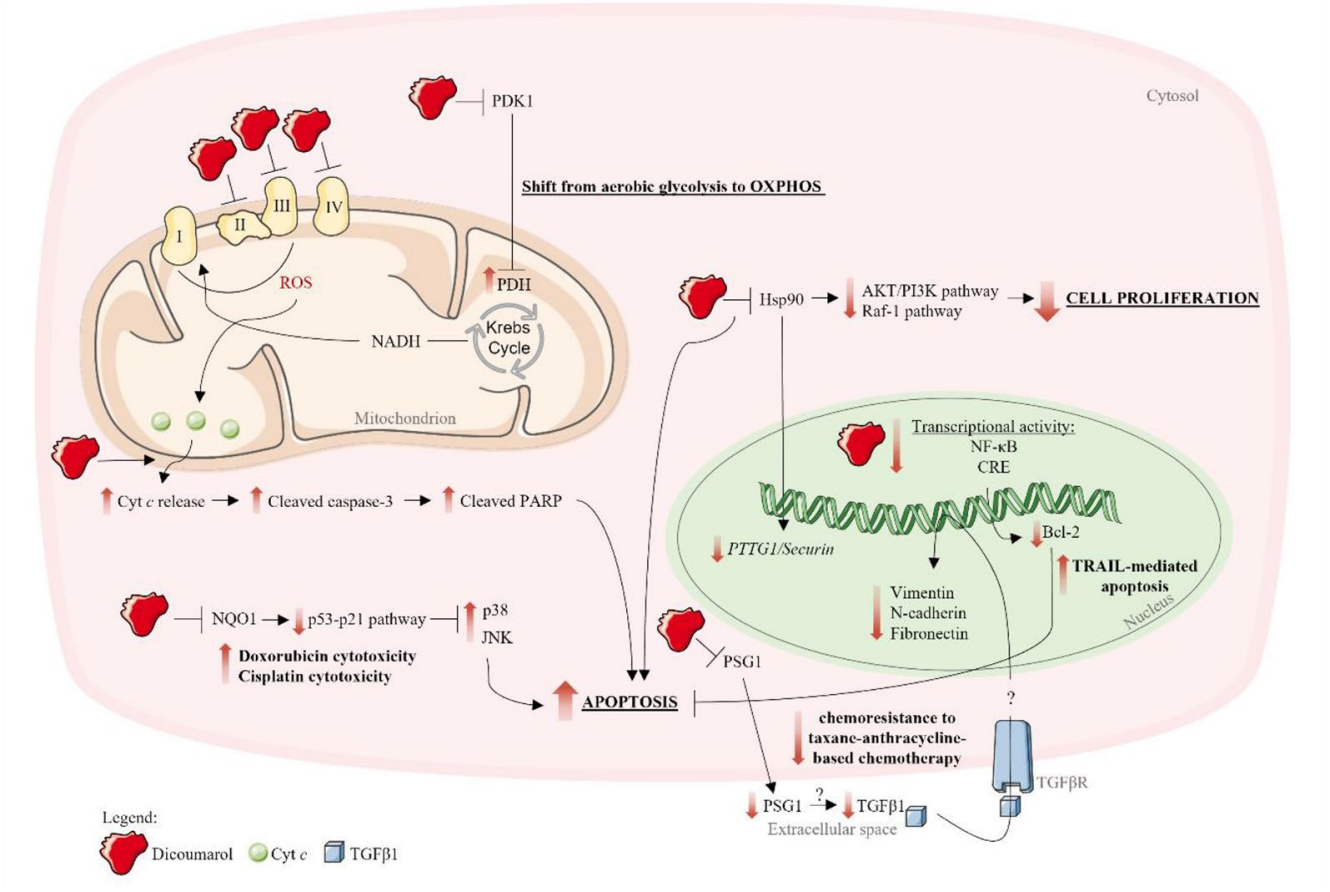
Actions of dicoumarol that may contribute to its antitumor effects. The figure was produced using *Servier Medical Art*. Legend—I, II, III, IV: oxidative phosphorylation complexes I, II, III and IV, respectively; cyt *c*: cytochrome *c*; CRE: cyclic adenose monophosphate response elements; JNK: c-Jun N-terminal kinase; NADH: nicotinamide adenine dinucleotide; NF-κB: nuclear factor kappa B; NQO1: NAD(P):(quinone acceptor) oxidoreductase 1; PARP: poly (adenosine diphosphate-ribose) polymerase; PDH: pyruvate dehydrogenase; PDK1: phosphoinositide-dependent kinase-1; PI3K: phosphoinositide 3-kinase; PSG1: pregnancy specific beta-1-glycoprotein 1; ROS: reactive oxygen species; TRAIL: Tumor necrosis factor-related apoptosis-inducing ligand; TGFβ1: transforming growth factor beta 1; TFGβR: transforming growth factor receptor

Another mechanism triggered by dicoumarol, independent of NQO1, seems to be the inhibition of PDK1, a key enzyme that negatively regulates the activity of the pyruvate dehydrogenase (PDH) complex by phosphorylation [103]. Inactivation of the PDH is associated with the Warburg effect (cancer cells rely on aerobic glycolysis—conversion of glucose to lactate even in the presence of oxygen—for energy production) [103, 108]. Dicoumarol inhibited the kinase activity of PDK1 in human ovarian cancer cells, shifting the glucose metabolism from aerobic glycolysis to OXPHOS (given the elevated glucose uptake and decreased lactate production), generating higher levels of ROS. Therefore, the mitochondrial membrane potential (MMP) was attenuated, inducing apoptosis, and reducing cell viability [103]. *In vivo*, dicoumarol was able to reduce tumor growth, possibly by targeting the kinase activity of PDK1, which induced the apoptosis of tumor cells [103]. Mechanistically, PDK1 inhibition results in an elevated influx of acetyl-CoA into the Krebs cycle, promotes NADH delivery to OXPHOS complex I, and induces an elevated production of ROS, which in turn damages redox-sensitive complex I and the proton pump [103]. Consequently, protons are incapable of efflux through the mitochondrial inner membrane, resulting in a decreased MMP (cancer cells exhibit a hyperpolarized MMP compared with normal cells) [103]. MMP depolarization opens the mitochondrial transition pore, resulting in the efflux of cytochrome *c* that makes part of the apoptosome. The pro-apoptotic proteins caspase-3 and PARP are then cleaved, leading to the activation of apoptosis, and therefore, to the suppression of tumor growth and reduction of cell viability both *in vitro* and *in vivo* [103]. Figure 4 overviews the main pathways regulated by dicoumarol in tumor cells.

Hitherto the majority of the data available on the effects of dicoumarol in cancer treatment are derived from *in vitro* studies. Therefore, preclinical studies are needed to validate data from *in vitro* studies and assess the putative therapeutic effects of dicoumarol, particularly, in targeting tumor growth and chemoresistance.

## Conclusions

The discovery of dicoumarol, a symmetrical natural biscoumarin, with important pharmacological activities, instigated numerous studies towards the development of efficient methods for its synthesis and of related derivatives, and investigation of their bioactivities. Among the synthetic methods, the most promising are those involving heterogeneous catalysis and the use of green solvents, which present economic advantages due to the possibility of recovering and reusing the catalyst and are also more sustainable.

In addition to its anticoagulant properties, dicoumarol has also gained a lot of interest due to other bioactive properties. Its anticancer properties are commonly associated to its ability to inhibit NQO1, but other effects, such as (i) impairment of mitochondrial functionality; (ii) decrease of PSG1 levels; (iii) inhibition of Hsp90; (iv) downregulation of anti-apoptotic proteins; and (v) inhibition of PDK1 may also occur. In recent years, a variety of cancer types, including colon, breast, ovarian, kidney, lung, urothelial, prostate, glioblastoma, and myeloid leukemia, have been shown to respond favorably to dicoumarol. However, although there is some* in vitro* evidence, further *in vivo* experiments and clinical trials are essential to establish the efficacy and safety of the mechanisms of action of dicoumarol and its derivatives. This review, devoted to dicoumarol and its derivatives, encloses the most relevant data related to their synthesis and mechanisms of antitumoral activity, which are crucial factors to reveal having in mind their future medicinal applications. Despite these stated evidences, further studies should be carried out to better understand the potential of dicoumarol derivatives in anti-cancer mechanisms, due to the lack of research in this area.

## Data Availability

Not applicable
